# A new interministerial strategy for the promotion of healthy eating in Portugal: implementation and initial results

**DOI:** 10.1186/s12961-018-0380-3

**Published:** 2018-10-30

**Authors:** Pedro Graça, Maria João Gregório, Sofia Mendes de Sousa, Sónia Brás, Tatiana Penedo, Telmo Carvalho, Narcisa M. Bandarra, Rui Matias Lima, Ana Paula Simão, Francisco Goiana-da-Silva, Maria Graça Freitas, Fernando Ferreira Araújo

**Affiliations:** 1 National Programme for the Promotion of Healthy Eating, Directorate-General of Health, Lisbon, Portugal; 2 Faculty of Nutrition and Food Sciences of University of Porto, Porto, Portugal; 3 EpiDoC Unit, Centro de Estudos de Doenças Crónicas (CEDOC) da NOVA Medical School, Universidade Nova de Lisboa (NMS/UNL), Lisbon, Portugal; 4 Technical Experts of the Cabinet of the Secretary of State for local Administration, Lisbon, Portugal; 5 Department of Sea and Marine Resources, Portuguese Institute for the Sea and Atmosphere (IPMA, IP), Rua Alfredo Magalhães Ramalho, 1495-006 Lisbon, Portugal; 6 Ministry of Sea, Lisbon, Portugal; 7 General-Directorate of Education, Lisbon, Portugal; 8 Legal Advisor to the Secretary of State of Industry, Lisbon, Portugal; 9 Department of Surgery and Cancer, Imperial College Medical School, London, United Kingdom; 10 Invited Assistant of Management and Leadership on Health, Health Sciences Faculty of Beira Interior University, Covilhã, Portugal; 11 Directorate-General of Health, Lisbon, Portugal; 12 Secretário de Estado Adjunto e da Saúde, XXI Governo Constitucional, Lisbon, Portugal; 13 Invited Professor of Faculty of Medicine of University of Porto, Porto, Portugal

**Keywords:** Food and nutrition policy, Portugal, Health in all policies

## Abstract

**Objective:**

To describe the implementation, main intervention areas and initial results of the Integrated Strategy for the Promotion of Healthy Eating (EIPAS) in Portugal.

**Methods:**

EIPAS was published as a Law, in December of 2017, as a result of a collaboration between several ministries, including the Finance, Internal Affairs, Education, Health, Economy, Agriculture, and Sea Ministries, aiming at improving the dietary habits of the Portuguese population. The working group, led by the Ministry of Health, developed this strategy for over a year. The framework produced was based on WHO and European Commission recommendations as well as on relevant data from the last Portuguese dietary intake survey (2015/2016). EIPAS also reflects the results of a public hearing, including the food industry, among others, and the experience gathered, since 2012, through the National Programme for the Promotion of Healthy Eating. It considers the ‘health in all policies’ challenge set by WHO and has four different strategic areas, namely (1) creation of healthier food environments, (2) improvement of the quality and accessibility of healthy food choices for consumers, (3) promotion and development of literacy, in order to encourage healthy food choices, and (4) promotion of innovation and entrepreneurship. In order to achieve these goals, a set of 51 actions was established and assigned to the seven ministries involved.

**Results:**

Under the scope of this strategy, Portugal has already implemented several actions, including (1) definition of standards for food availability at all public healthcare institutions; (2) implementation of a sugar tax on sweetened beverages; (3) implementation of a voluntary agreement with the food industry sector for food reformulation (work in progress); (4) design of a proposal for an interpretative model of front-of-pack food labelling; (5) improvement of the nutritional quality of food aid programmes for low-income groups; and (6) regulation of marketing of unhealthy foods to children.

**Conclusions:**

For the first time, Portugal has a nutrition policy based on the WHO concept of ‘health in all policies’ and on the national data on food intake. The implementing process of all 51 actions and the inherent complexities and difficulties found so far have made this process be an authentic political and social laboratory that deserves to be followed.

## Background

Unhealthy dietary habits are one of the main risk factors for the burden of disease in Portugal. According to data from the Global Burden of Disease study 2015, 15.8% of the disability-adjusted life years in Portugal are due to an unhealthy diet [1]. The prevalence of overweight in Portugal is high in adults and children. More than 50% of the adult Portuguese population is overweight (including obesity, body mass index ≥ 25 kg/m^2^) [2] whereas, for children aged between 6 and 8 years, this indicator reaches 30% (using body mass index-for-age according to WHO classification), categorising Portugal as one of the countries with the highest prevalence of child overweight in Europe [3]. Additionally, the growing burden of non-communicable diseases is one of the main public health challenges in Portugal. According to the Global Burden of Disease study 2015, 86% of the total burden of disease is due to non-communicable diseases. In 2014, cardiovascular diseases were the leading cause of death in Portugal, and stroke was more prevalent in Portugal than in all other European countries [1, 4]. Moreover, poverty and social inequalities, which are important issues in Portugal [5], are closely linked to unhealthy diets and to diet-related chronic diseases. Data from the last national health survey showed a higher prevalence of several chronic diseases in the lower educational level groups (diabetes: 12.2% in individuals with less than 4 years of education vs. 6.4% in individuals with more than 12 years of education; hypertension: 45.1% in individuals with less than 4 years of education vs. 25.6% in individuals with more than 12 years of education) [6]. The same pattern is observed for obesity (38.5% in individuals with less than 4 years of education vs. 13.2% in individuals with more than 12 years of education) [7]. Despite these data not being age-standardised, other studies previously published in Portugal, which have used adjusted models, have shown the same social gradient [8].

Considering this epidemiology context, in 2012, Portugal implemented the first national food and nutrition policy – the National Programme for the Promotion of Healthy Eating (PNPAS). PNPAS was approved by Order n.° 404/2012 of January 3, 2012, having been considered one of the eight priority programmes to be developed by the Ministry of Health [9]. PNPAS was created to fulfil the mission of improving the nutritional status of the population, stimulating the physical and economic availability of healthy foods and creating conditions so that the population can value, appreciate and integrate them into their daily routines [10, 11].

**Fig. 1:**
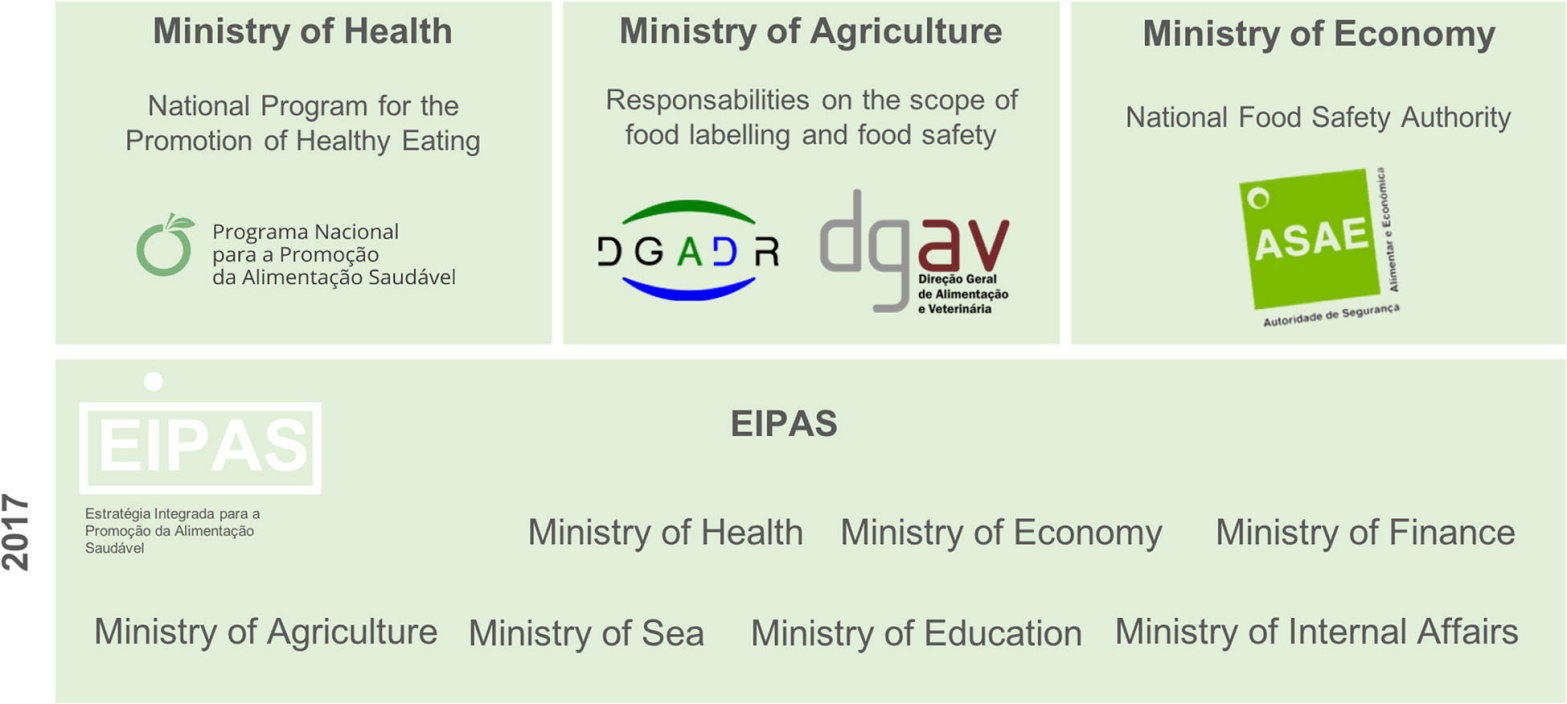
Organisation of the food and nutrition field in Portugal

In terms of policy structure, Portugal is a Republican state and a parliamentary democracy with a centre-left government since November 2015. Three levels of governance exist in Portugal – central, regional (Autonomous Regions) and local level (municipalities), with the legislative responsibilities falling under the scope of the central level. In Portugal, the nutrition policies are under the responsibility of the Ministry of Health, with the exception of food labelling issues, which fall under the scope of the Ministry of Agriculture. Several public technical institutions, such as the Directorate-General of Health, support the Portuguese government on policy formulation, implementation and evaluation. The Directorate-General of Health is a public body of the Ministry of Health, which plans, regulates, coordinates and supervises all health promotion activities, including food and nutrition programmes. However, other important areas on food and nutrition fall under the scope of other ministries (Fig. 1).

During the first couple of years of implementing the PNPAS, several actions were undertaken at different levels. However, an important challenge for food and nutrition policies remains in the difficulty of targeting other sectors when a policy is mainly promoted and executed by the health sector. This challenge is related to the necessity of having a broad multi-sectoral approach, establishing alliances and partnerships, namely among the different government sectors, to create healthy food environments. In fact, the need for a more intense intersectorial approach is currently one of the main challenges for food and nutrition policies, especially in countries like Portugal, where integrated policies are not common.

To overcome the challenge of ‘health in all policies’ or of a ‘whole-of-government approach’, in 2016, the Portuguese government created a workgroup to develop an interministerial strategy for the promotion of healthy eating. After 1 year of intense work, the Integrated Strategy for the Promotion of Healthy Eating (EIPAS) was initiated by Order n.° 11,418/2017 of December 29, 2017 [12].

The purpose of this paper is to describe the design and implementation process, main intervention areas and initial results of the new EIPAS in Portugal.

## Methods

### Agenda setting/identification of problems and issues (Diagnosis)

The first national programme on food and nutrition in Portugal (PNPAS) was launched in 2012. This programme was established as one of the eight priority national programmes of the Directorate-General of Health and has five general objectives, namely to (1) increase knowledge on food consumption of the Portuguese population, its determinants and consequences; (2) change the availability of certain food products in public settings; (3) inform and empower individuals on the purchase, preparation and storage of healthy foods; (4) identify and promote cross-sectional actions that encourage the consumption of good nutritional quality foods, in an articulate way with other sectors; and (5) improve qualifications of the different professionals who, owing to their roles, may influence nutritional knowledge, attitudes and behaviours. Several actions, at different levels, were implemented during the first couple of years. One of the main achievements of this programme was the development of a new communication strategy to disseminate accurate information on food and nutrition. Digital channels, such as the blog (Nutrimento Blog at www.nutrimento.pt) and the PNPAS Website (www.alimentaosaudavel.dgs.pt), and the use of social networking sites (Twitter and Instagram) have been considered the preferential channels to spread information, making them a reference nationwide. The communication strategy has also included the production of podcasts and reference manuals (posters, guidelines), both for professionals and health and education institutions. Furthermore, the amount of available data regarding nutritional status, food consumption, their determinants and health outcomes across the lifecycle has been considerably increased [13]. PNPAS has developed a set of actions aiming to directly collect or aggregate information at several levels, namely supporting (scientifically and/or financially) the development of research projects; for example, it (1) implemented a monitoring system for childhood obesity; (2) developed a study about consumers’ understanding of nutrition labelling; (3) studied the presence of trans fatty acids in the Portuguese food market; (4) implemented a surveillance system on food insecurity in Portugal; (5) developed a project to map and disseminate good practices in community intervention projects for obesity prevention; and (6) gave technical support for the development of the national food and nutrition survey 2015, after a period of more than 40 years without information regarding the food consumption of the Portuguese population. The data obtained, through the PNPAS effort, was determinant for the policy planning of the EIPAS. Between 2012 and 2016, several partnerships were established with various stakeholders. Several institutions from the public and private sectors as well as civil society were involved. However, the first few years of implementation of the PNPAS were characterised by difficulties in obtaining a formal whole-of-government commitment regarding the Public Health Programme Goals. Therein, we identified several policy disparities between different Government sectors, compromising the successful implementation of several interventions, in particular those related to the promotion of healthier food environments.

Therefore, in order to align and potentiate all the different approaches regarding the area of nutrition at a national level, a better articulation between Ministries in the Portuguese Government was required.

### Policy formulation (planning)

In this section, we will describe the implementation process of the EIPAS.

On September 15, 2016, the Portuguese Council of Ministers, through the Deliberation of the Council of Ministers no. 334/2016, determined the creation of an interministerial working group on the promotion of healthy eating. This working group was created in order to elaborate the EIPAS, gathering contributions and commitment from all Ministries with responsibilities in the area of nutrition [14]. The EIPAS’ main objectives were to encourage healthier food consumption habits as well as to improve the nutritional status of the Portuguese population [14]. EIPAS followed the recommendations of WHO for an integrated approach on ‘health in all policies’, fitting into one of the strategic axes of the National Health Plan (revision and extension to 2020), particularly the Public Health axis, based on the premise that everyone should contribute to the creation of health-promoting environments and the well-being of the population. This strategy was designed to address three main issues, namely (1) to modify the supply of certain foods, particularly those with high sugar, high salt and high fat content; (2) to encourage actions of nutritional reformulation of food products through an articulated action with the food industry, food distribution and also with the food and beverage service providers; and (3) to empower citizens and professionals who work on or influence the consumption of food to encourage healthy food choices.

Seven different ministries composed the working group, namely the ministries of Finance, Internal Affairs, Education, Health, Economy, Agriculture, and Sea. This working group, led by the health sector, worked for over a year, holding monthly meetings. As a result, the group identified priority areas, challenges and opportunities for intervention. Following 6 months of work since the first meeting of this working group (5 December 2016), the first draft of the strategy was sent to the Government.

The framework produced was based on the recommendations from the WHO European Food and Nutrition Action Plan 2015–2020 and from the High Level Group on Nutrition and Physical Activity of the European Commission (DG Santé, European Commission). The previous experience of the Norwegian action plan on nutrition, as the first integrated policy on nutrition and involving 12 ministries, was also used as a reference [14], as well as the last Portuguese dietary intake survey (2015/2016) and other recent studies in the field. The analysis of these recent data allowed the identification of the main nutritional problems of the Portuguese population, creating different proposals for intervention.

The cycle of conferences on the ‘Future of Food’ organised by the Calouste Gulbenkian Foundation, during 2012, in which several national and international experts from different fields were invited to discuss the future challenges of food production and consumption, also contributed to the EIPAS design. Various perspectives were also analysed in order to achieve the common goal, namely of achieving “*people’s health and well-being, environmental sustainability, and equitable access to food for all while enhancing economic development prospects via added value and job creation*” [15]. The final discussions and conclusions brought together dozens of experts who have identified the main challenges and needs in the construction of a national food policy, without losing its ability to be opened to the growing globalisation of world food circuits.

On the Council of Ministers deliberation no. 334/2016, on September 15, 2016, four strategic areas of intervention and proposals of different initiatives/actions related to each strategic axis were defined based on the proposals submitted by the different ministries involved in the EIPAS working group.

Following strategy approval by the Portuguese Government, a public consultation was launched (August 2017) in order to collect the views of all interested parties. Thus, EIPAS also reflects the results of a public hearing from several stakeholders, including the Portuguese Food Industries Association, the Portuguese Food Distribution Association, the Portuguese Catering Association, the Portugal Agricultures Association, the National Confederation of Agriculture, the National Confederation of Young Agricultures, the Portuguese Business Confederation, the National Association of Advertisers, the National Association of Portuguese Municipalities, the National Association of Portuguese Parishes, the Portuguese Association for Consumer Protection and the Portuguese College of Nutritionists. Inputs from WHO and the Food and Agriculture Organization were also collected as well as from some Portuguese academic and research institutions.

Following the suggestions and recommendations obtained through the public consultation, the strategy was revised by the working group and, in October 2017, the final proposal was sent to the Government.

**Fig. 2:**
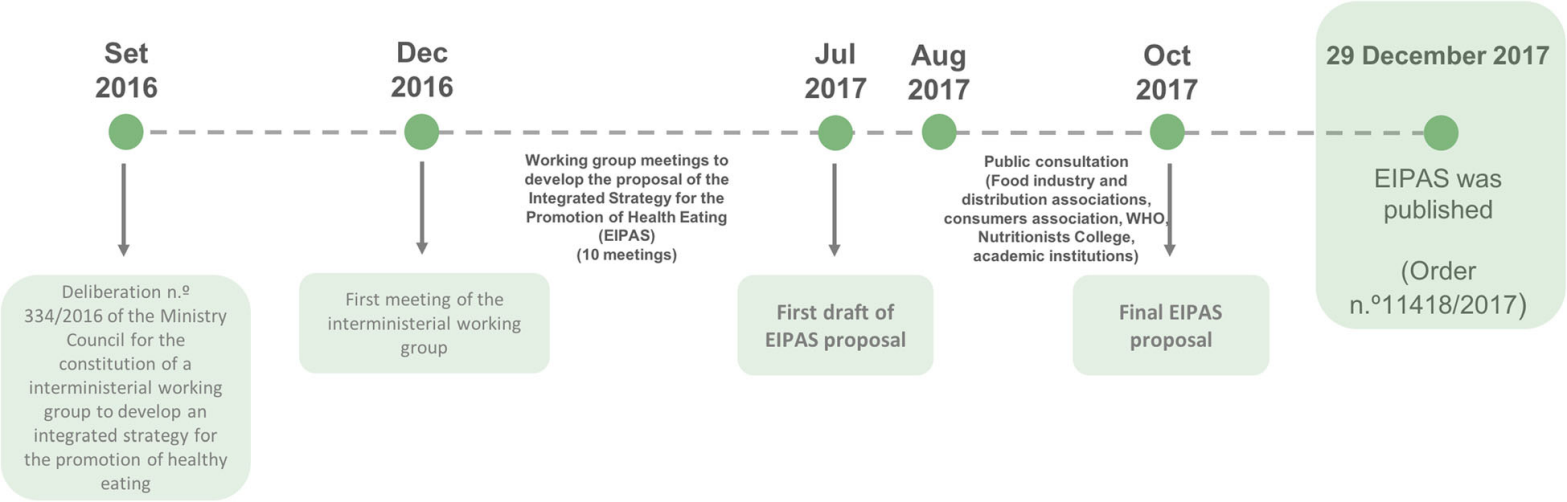
Timeline of the implementation process of EIPAS

After 1 year of work, EIPAS was published as a law by Order n.° 11,418/2017 of December 29, 2017 (Fig. 2). The Directorate-General of Health was made responsible for the follow-up and monitoring of the implementation process. In order to do so, the EIPAS working group remained the same to allow further articulation among all its members under the coordination of PNPAS.

The uniqueness of EIPAS lies in the fact that it has set goals for the promotion of healthy eating through the participation of six ministries other than the Ministry of Health. These ministries and sectors, such as the Finance, Economy and Local Authorities, are not usually involved in these areas of public policies. On the other hand, the general proposals of political intervention, such as changing consumer preferences or choice, food reformulation, and food availability and accessibility, are central, but the connection to economic growth is also considered. The EIPAS strategy does not separate the promotion of healthy eating habits from the promotion of innovation and entrepreneurship (see below strategic area 4). Finally, several measures aim to promote a more equitable food system, which is rare in traditional nutritional policies.

### Strategic areas and main intervention areas

EIPAS has four strategic intervention areas, namely (1) to create healthier food environments by modifying the types of food provided or sold in different public settings and promoting the reformulation of certain categories of food that are high in salt, sugar and fat, (2) to improve the quality and accessibility of healthy food choices for consumers in order to inform and empower citizens to make such choices, (3) to promote and develop consumer literacy for healthy food choices, and (4) to promote innovation and entrepreneurship focused on the area of promotion of healthy eating. Different actions were defined for each strategic area, reaching a total of 51 actions.

### Strategic area 1 – create healthier food environments

The objective of the first strategic area of EIPAS is to modify the environment where people choose and buy food by modifying the food that is provided or sold in certain public settings and promoting the reformulation of certain food categories. This strategy aims to make healthy food choices easier in terms of price, access and attractiveness, promoting the improvement of the availability and composition of foods, particularly with regards to their salt, sugar and trans fatty acid contents. This strategic area is composed by four main priority areas of intervention, namely (1) monitoring the nutritional composition of food, (2) implementation of initiatives on food product reformulation, (3) improvement of food availability in different public settings, and (4) improvement of food availability in the catering sector. In order to achieve these goals, 20 different actions were proposed (Table 1).

**Table 1:** Actions for Strategic area 1 (create healthier food environments)

Strategic area 1 – Create healthier food environments	
Main areas of intervention	Actions
Monitoring the nutritional composition of foods	To monitor the salt content in the following food categories: bread and breakfast cereals, meat and meat products (minced meat, meat product), ready-to-eat meals, fried potatoes and other snacks, sauces, ready-to-eat soups, cheeses, canned fish and restaurant meals (soup and main dish) [29, 31]
	To monitor the sugar content in the following food categories: non-alcoholic beverages, dairy products, cookies and sweet desserts, pastries, breakfast cereals, ready-to-eat meals, sauces, ice cream and canned fruit [30, 31]
	To monitor the trans fatty acids content in the following food categories: biscuits, pastries, fried potatoes, breakfast cereals, chocolate spreads and margarine [31, 44]
Food products reformulation	To promote the adequacy of nutrient profiles for certain categories of foods, particularly for salt, sugar and trans fatty acids content: 1.1 – For food products, it is intended to achieve the values set out in Regulation (European Commission) no. 1924/2006 of the European Parliament and of the Council of December 20^th^, 2006, on nutrition and health claims made on foods, which defines foods with a low salt content as not containing more than 0.3 g of salt per 100 g or 100 mL; 1.2 – For soup and main dishes it is defined that the amount of salt should be lower than the reference value of 0.2 g of salt per 100 g of food. This reference value agrees with that defined in the document “Strategy for reducing the salt intake in the Portuguese population by modifying the availability of the food availability”, approved by the inter-ministerial working group to propose a set of measures for the reduction the salt consumption by the Portuguese population; 1.3 - For food products, it is intended to achieve the values set out in Regulation (European Commission) no. 1924/2006 of the European Parliament and of the Council of December 20^th^, 2006, on nutrition and health claims made on foods, which defines a food with a low sugar content as not containing more than 5 g of sugar per 100 g for solid foods or 2.5 g of sugar per 100 mL for liquids [29–31, 59]; 1.4 – For industrial fats sold for the manufacture of foods, as well as in the final product, a trans fatty acid content of not higher than 2 g per 100 g must be established [34]
	To propose goals to be achieved in the reformulation of categories of food described above along with sector entities, based on the WHO Recommendations [60, 61] and as a general goal: 1.1 – To make the per capita salt intake close to 5 g/day by 2020; 1.2 – To make the daily sugar intake close to 50 g/day and a maximum of 25 g/day for minors by 2020; 1.3 - To make the trans fatty acid intake close to zero by 2020
Change food availability in different public settings	To extend Dispatch no. 7514-A/2016, published in *Diário da República*, 2nd series, no. 108, of 6th June 2016, to all services and organisms of the direct and indirect administration of the State about new contracts regarding vending machines services
	To propose the existence of free water dispensers or distribution of water in the services and organisms of the direct and indirect administration of the State and in other services under public management and to promote their consumption [62, 63]
	To propose the obligatory availability of water, fruit and vegetables, preferentially respecting criteria of seasonal availability and proximity, at public events organised by the services and organisms of the direct and indirect administration of the State
	To extend the existing guidelines for the provision of food in schools from the Ministry of Education, to all levels of education, including higher education
	To establish guidelines for the supply of food at social services institutions, particularly those who support the elderly population
	To encourage public institutions to purchase food products that use short distribution channels, integrated or organic production modes. To this end, it is proposed to produce a guide containing clear indications about the award criteria and the factors and sub-factors associated. This guide should bring the producer and the consumer closer, namely in the food services supervised by services and organisms of the direct and indirect administration of the State
	To extend the Directorate-General of Education (DGE) school canteen guidelines regarding the use of iodised salt to other cafeterias/canteens beside those in schools
	To encourage fruit and vegetable intake at schools, increasing the number of beneficiaries of the school distribution scheme, which includes the current fruit and vegetable distribution schemes and the school milk distribution scheme
Change food availability at food industry and catering sector	To encourage the consumption of food categories directly related to the prevention of chronic disease, namely fresh fruit and vegetables, across the population
	To encourage the removal of salt shakers from tables in collective catering establishments, proposing that salt shakers must be dispensed only by explicit request of the costumer
	To encourage and extend the scoop of good practice related with DGE school cafeteria guidelines in the context of new calls for food procurement for school meals
	To encourage agro-food companies to reduce the size of pre-packaged food and drinks
	To encourage catering services to provide adapted menus for the most prevalent diseases
	To extend policies to limit the volume and availability of individual sugar packages to all economic agents responsible for the refining and distribution of sugar
	To encourage catering services to avoid the availability of sugar beverages in the ‘free refill’ mode

### Strategic area 2 – improve quality of and consumer accessibility to healthy food choices

EIPAS’ second strategic area aims to improve the quality and accessibility of available information for consumers in order to inform and empower citizens to make healthy food choices. This strategic area is intended to identify activities/initiatives that facilitate citizens’ access to quality information for informed and responsible choices. Nowadays, there are large amounts of information regarding food and nutrition, yet it is difficult for citizens to distinguish the reliable information provided by independent and credible authorities, like the State, which should play an important role in this matter. For this strategic area, five priority intervention areas were identified, namely (1) improvement of nutrition labelling on food, (2) restriction of marketing of unhealthy foods to children, (3) promotion of communication about food and nutrition in public institutions, (4) implementation of local strategies for the promotion of healthy eating, and (5) dissemination of good practices for healthy eating promotion. In order to achieve these goals, 11 different actions were proposed (Table 2).

**Table 2:** Actions for Strategic area 2 (improve quality of and consumer accessibility to healthy food choices)

Strategic area 2 – Improve quality of and consumer accessibility to healthy food choices	
Main areas of intervention	Actions
Nutrition labelling on food	To encourage the use of additional nutritional information in food labelling in order to facilitate consumer’s food choices, namely by producing guidelines for the food sector operators
	To encourage the use of information regarding the trans fatty acid content as part of the nutritional information in food labelling
	To promote a public consumer information campaign about healthy eating, including information on nutritional labelling
Marketing to children of unhealthy foods	To encourage the adoption of measures by economic operators to restrict the marketing to children of food products high in salt, sugar, fat – namely trans fatty acids – and energy
	To encourage the adoption of measures by economic operators in order to limit commercial communication and advertising of food products with high amounts of salt, sugar, fat – namely trans fatty acids – and energy, during events involving underage people, such as sports, cultural, recreational and other activities
Communication about food and nutrition in public institutions	To use digital media to promote quality messages about healthy eating
	To developed initiatives in partnership with the agro-food sector with the aim of providing information about healthy eating at the points of sale
	To promote the presence of short and simple messages about healthy messages in the periodic documents for the general public by the ministries involved in the working group, whenever possible
Local strategies for the promotion of healthy eating	To promote the involvement of local authorities to provide information about healthy eating through their own tools and methods
	To promote the inclusion of healthy eating promotion initiatives in public health, physical activity and active aging promotion programmes of municipals
Dissemination of good practices for healthy eating promotion	To develop a platform for disseminating and monitoring the initiatives foreseen in this Integrated Strategy for the Promotion of Healthy Eating

### Strategic area 3 – promote and develop literacy for consumers’ healthy food choices

This strategic area intends to promote and develop literacy and autonomy for consumers to make healthy choices, enabling citizens of various levels of literacy to make such choices. In order for this programmatic area to succeed, it is necessary to involve and train professionals, other than those involved in health, to promote healthy eating habits in the populations they work with. This strategic area includes four priority areas, namely (1) improvement of food and nutrition literacy from the paediatric age, (2) promotion of the Mediterranean diet with the traditional Portuguese emphasis on seafood, (3) improvement of communication on food and nutrition for the general population, and (4) empowerment and increased knowledge on nutrition of different professionals. In order to achieve these goals, 15 different actions were proposed (Table 3).

**Table 3:** Actions for Strategic area 3 (promote and develop literacy for healthy consumer food choices)

Strategic area 3 – Promote and develop literacy for healthy consumer food choices	
Main areas of intervention	Actions
Improve food and nutrition literacy at paediatric age	To promote food literacy of pregnant women and parents about the importance of healthy eating in the first 1000 days of life
Promote Mediterranean diet	To promote strategies of healthy food education in the school environment, in particular through the promotion of the Mediterranean diet, preparation and cooking of food, and better knowledge of the food production cycle
	To promote initiatives that value the knowledge about nutritional value of strategic and characteristic foods present in the Mediterranean Food Wheel, namely fish, fruits and vegetables, olive oil, bread, pulses and dairy products
	To promote initiatives that value the consumption of autochthonous breeds/varieties and typical food of the Mediterranean diet. In particular, herbs and/or species should be valued as substitutes for salt, and salicornia plant as an alternative to salt
	To increase the citizen’s knowledge about the concept of Mediterranean diet and how to promote healthy choices from this model
Improve communication on food and nutrition for the general population	To developed additional supplementary measures to the distribution of milk and vegetables in schools, enhancing the efficacy of the school distribution system in promoting eating healthy habits
	To promote initiatives to raise public awareness about the health impact of excessive intake of salt, as well as initiatives that promote the use of salt substitutes such as herbs and spices, and salt alternatives like salicornia
	To promote initiatives to raise public awareness about the health impact of excessive sugar intake
	To promote initiatives that encourage healthy eating among the university population
Empowerment and increased knowledge on nutrition of different professionals	To improve the qualification of social services’ professionals in the healthy eating area, particularly those in contact with people with lower levels of income and literacy and who are responsible for the management of food products distribution.
	To promote the involvement of municipalities and parishes in the implementation of eating healthy training schemes for the workers of food services and cafeterias
	To improve the qualification of professionals in the tourism and catering area about healthy eating, particularly regarding the risks of excessive salt, sugar and trans fatty acid intake
	To increase the knowledge of food service and cafeteria workers about how to incorporate fish, vegetables and fruits into the elaboration of menus and meals
	To capacitate different healthy professionals on the importance of sensitising parents to the importance of breastfeeding [63]
	To capacitate healthy professionals, teachers and tutors to promote taste and preference for healthy food in whilst young [63]

### Strategic area 4 – promote innovation and entrepreneurship focused on the area of healthy eating promotion

The aim of this strategic area is to identify initiatives that use innovation and technological development to change knowledge, attitudes and behaviours towards healthy eating, taking as an advantage the entrepreneurial capacity of the Portuguese economy and small-scale companies. This strategic area includes three priority areas of intervention, namely (1) utilisation of digital communication for the promotion of healthy eating, (2) reorientation of research on financial priorities, and (3) promotion of monitoring systems for food consumption and nutritional composition. In order to achieve these goals, five different actions were proposed (Table 4).

**Table 4:** Actions for Strategic area 4 (promote innovation and entrepreneurship focused on the area of promotion of healthy eating)

Strategic area 4 – Promote innovation and entrepreneurship in the area of promotion of healthy eating	
Main areas of intervention	Actions
Use digital communication for the promotion of healthy eating	To build an online information platform about fish and its nutritional value, providing interactive materials for teaching, and to support the presence of fish in school meals
	To promote the use of digital media in public institutions (e.g. waiting rooms, service desks) for the promotion of healthy eating
Reorient research financial priorities	To propose alignment of funding priorities for research, in state laboratories and funding agencies, with the national priorities in the area of healthy eating promotion
Monitoring systems for food consumption and foods nutritional composition	To propose the creation of innovative and sustainable monitoring systems that allow a permanent evaluation of food intake
	To allow the access to free and universal nutritional information of food products through a digital platform, which will be publicised among the general population, in order to stimulate the creation of new entrepreneurial initiatives that promote healthy eating

## Results

### Policy implementation (output) – Portuguese food and nutrition strategy: a short overview of the main actions already implemented

Under the scope of the EIPAS and the work previously done by the PNPAS and the Ministry of Health, several actions have already been implemented, including (1) the definition of standards for food availability at public settings; (2) the implementation of a sugar tax on sweetened beverages; (3) improvement of the nutritional quality of the national food aid programme for low-income groups; (4) implementation of a voluntary agreement with the food industry sector for food reformulation; (5) design of a proposal for an interpretative model of front-of-pack food labelling; and (6) regulation of marketing of unhealthy foods to children.

#### Implemented actions on the improvement of food available in different public settings (Strategic area 1 – create healthier food environments)

1. Healthy food procurement policies or standards for food availability in public settings (schools and healthcare facilities)

Governments should be committed to the promotion of healthy food environments for all citizens, allowing them to make healthier food choices. According to a recent review conducted by Raine et al. [16], healthy food procurement policies can have a positive effect in several outcomes such as on improving food availability and on increasing sales and intake of healthy food options. In the school environment, the Portuguese Ministry of Education and the Ministry of Health have had a good collaboration experience. Mandatory nutrition standards for school lunch meals were established in 2013 [17], advocating, for example, the use of iodised salt in schools. A guide for food supply in school buffets/cafeterias and vending machines was also developed, aiming to support schools in selecting the best food options [18]. Based on this work as well as on previous work in some Regional Health Administrations, the Portuguese government introduced mandatory food standards for vending machines and buffets/cafeterias in healthcare facilities during 2016 and 2017 [19, 20].

2. The Portuguese sugar tax on sweetened beverages

In February 2017, Portugal introduced a sugar tax on sweetened beverages. Considering the experience on food price policies implemented in other countries and a scientific report published by the Directorate-General of Health [21], this strategy aimed to encourage food reformulation. The tax mechanism used in Portugal was specific excise taxing, wherein a tax of 16.46€ per hectoliter is applied for soft drinks that contain ≥ 8 g of sugar per 100 mL and a tax of 8.22€ is applied for those with < 8 g of sugar content per 100 mL. Concentrated fruit juices and milk products were excluded from this tax [22]. The funds obtained by this tax were to be used to finance public health interventions. This tax generated controversy among the food industry sector, yet public opinion was, in general, positive.

No formal evaluation has been conducted to analyse the infer causality of this tax so far; however, preliminary results of the impact of this strategy suggest a reduction of almost 50% in the consumption of sweetened beverages with ≥ 8 g of sugar per 100 mL and, further, that this reduction has contributed to a decrease of 15% in total sugar consumption from these food products (more than 5600 tons of sugar) [23]. We think that these results reflect the ensuing product reformulation. However, with these data, it is not possible to identify if these results are also due to the reduction of consumption of these food products.

3. The new food aid programme for low-income groups in Portugal

Food insecurity is a public health problem in Portugal. According to a study conducted in a cohort of the Portuguese population, food insecurity has a prevalence of 19.3%. Additionally, this study has shown that Portuguese food-insecure individuals have a higher risk of having unhealthy dietary patterns (lower adherence to the Mediterranean diet) and chronic diseases, resulting in higher healthcare resource consumption [24]. Data from the last National Health Survey showed a social gradient in the prevalence of chronic diseases such as diabetes, hypertension and obesity. The prevalence of these chronic diseases is two or three times higher in the lower educational level groups compared to their aged-matched counterparts [6]. These social inequalities in health can be partly attributed to those observed in the modifiable risk factors for chronic diseases, including an unhealthy diet.

Food aid programmes are interventions commonly used to deal with food insecurity, yet these interventions do not always have the capacity to reduce diet- and health-related social inequalities. Various studies have shown that this kind of programmes can considerably reduce these inequalities, yet several countries, including European countries, fail in their food aid programmes due to their low capacity to provide nutritionally adequate food [25].

In 2016, PNPAS established a protocol together with the Ministry of Social Security aiming to help the development of a new strategy for the Portuguese food aid programme for the most deprived. This strategy was developed under the Fund for European Aid to the Most Deprived of the European Commission and was based on three main pillars, namely (1) development of nutritional guidelines for the Portuguese Food Basket for the most deprived [26], (2) empowerment of social institutions responsible for the food basket distribution, and (3) improvement of food and nutrition literacy and cooking skills of the beneficiaries [27, 28]. Nutritional guidelines for food baskets were developed for five different age groups – children aged between 5 and 11 years, children/adolescents/young adults aged between 12 and 28 years, adults aged between 19 and 60 years, and adults aged over 60 years. The food baskets developed were composed by 18 different foods, representing all the food groups of the Portuguese food guide, in the right proportions, with the exception of fruit. These food baskets were distributed monthly and ensured 50% of the daily nutritional requirements. The main concerns for the development of these food baskets were the need to increase the distribution of vegetables and protein source foods (meat and fish) as well as to decrease the distribution of energy-dense foods with high levels of sugar, salt and fat [26].

#### Implemented actions in terms of initiatives on food product reformulation (Strategic area 1 – create healthier food environments)

1. The voluntary agreement with the food industry for food reformulation

To achieve healthier food environments, it is essential to promote a collaborative approach with the food industry. We started this work in January 2018 and it is currently on course. We are now establishing targets for levels of salt and sugar for some selected food categories. This work is being performed by a working group coordinated by the Directorate-General of Health and composed by the Portuguese Food Industries Association, the Portuguese Food Distribution Association, the Portugal Foods Association, the Portuguese Association for Consumer Protection, the Portuguese Nutrition Association, and the Portuguese Association of Nutritionists.

The main food categories were already identified and are aligned with the recommendations of the High Level Group on Nutrition and Physical Activity from the European Commission [29–31]. For the food categories selection, we also considered the data from the last national food consumption survey regarding the foods that most contribute to the sugar and salt intake in the Portuguese population, and in particular in children [7]. The target levels of salt and sugar will also be defined based on the recommendations of the European  Commission and based on the experience of the United Kingdom [32, 33]. These targets will also be defined considering the data of the nutritional composition of the available foods in the Portuguese market, in particular of those that represent more than 85% of volume in sales. Seven categories of food were identified with regards to salt (toast, breakfast cereals, cookies/biscuits, ham, cheese, salted snacks, ready-to-eat soup) and six were identified for sugar (yogurt, milk with chocolate, breakfast cereals, cookies/biscuits, soft drinks and concentrated fruit juices). The general goal for salt and sugar consumption is to achieve a reduction of 16% and 10–20% by 2021, respectively. Besides the identification of these top priority categories of foods, this voluntary agreement will also encourage all sectors of the food industry to get involved in this reformulation process.

This voluntary agreement also includes a strategy to reduce trans fatty acids in Portuguese foods by defining a limit of 2 g per 100 g of fat in margarine/shortening according to WHO recommendations [34].

One of the food categories that most contributes towards salt intake is bread. Since 2009, Portugal has implemented a law that establishes the maximum limit of salt in bread to no more than 1.4 g of salt per 100 g of bread [35]. However, in October 2016, a voluntary agreement with the Portuguese Association of Bakery Manufacturers was established to reduce this limit to a maximum level of 1 g of salt per 100 g of bread, to be achieved by 2021.

#### Implemented actions in terms of initiatives to improve nutrition labelling on food (Strategic area 2 – improve quality of and consumer accessibility to healthy food choices)

1. Interpretative model of a front-of-pack nutrition labelling

PNPAS has been working on the development of an interpretative model of a front-of-pack nutrition label. However, it has been a difficult task since a consensus between the different stakeholders on this subject has not yet been reached. In 2014, PNPAS and the Directorate-General of Health presented a proposal for a guide to use traffic light nutrition labelling, but it was not approved. In 2017, PNPAS supported the development of a study with the technical support of WHO Europe in order to provide additional knowledge for policy decision-making, namely knowledge about Portuguese consumers’ understanding, preferences and usage of different front-of-pack models. Data from this study showed that 40% of the Portuguese consumers did not understand the current nutrition labelling model and this proportion increased to 60% when considering Portuguese consumers with a low educational level. Moreover, this study showed that a scheme like traffic light labels is one of the most preferred choices [36]. After the publication of this report, two government political parties presented a proposal for the application of traffic light nutrition labelling in Portugal [37, 38]. However, these proposals were rejected and the Portuguese Parliament has recommended the government to study an alternative model [39].

#### Implemented actions in terms of initiatives to restrict marketing of unhealthy foods to children (Strategic area 2 – improve quality of and consumer accessibility to healthy food choices)

1. Regulation of unhealthy food marketing to children

Since 2016, PNPAS coordinates the WHO European Action Network on Reducing Marketing Pressure on Children, aiming at finding ways to reduce marketing pressure on children regarding energy-dense, micronutrient-poor foods and beverages. In 2016, a new law was proposed with restrictions on advertising towards children regarding food and drinks in Portugal. The law now has general approval from the three political parties that support government. The law forbids the advertising of food and beverages high in sugar, fat or salt within the 30 minutes previous and subsequent to and during children’s television programmes as well as for TV programmes whose audiences have a minimum of 20% audience younger than 12 years old as well as on the internet or web pages with content intended for children and within a surrounding radius of 500 m from schools [40].

EIPAS is less than 6 months old. The specific and detailed measures have been explained and its execution rate is being monitored in the 51 proposals. As a national government initiative involving many stakeholders, many of the initiatives in full implementation were not described in detail. For example, a discussion with various sectors of the food industry is currently ongoing regarding the reformulation of food products, but given its secretive, unfinished and uncertain nature, it is difficult to describe.

### Assessment/evaluation (outcome/impact)

The EIPAS working group is responsible for monitoring the implementation progress of this strategy and for delivering biannual reports to the Portuguese Government. All the actions implemented under the EIPAS scope should include an impact assessment.

## Discussion

### The challenge of a food and nutrition policy to tackle non-communicable diseases

In order to address the public health problem of non-communicable diseases (the main cause of morbidity, disability and mortality) a new health policy approach with a focus on efficient prevention is necessary. Within this approach, the need for the promotion of a healthy diet emerges as a top priority. Unhealthy dietary habits are closely linked to the most prevalent chronic diseases, and represent the main risk factor for the burden of disease in Portugal [1]. In recent years, nutrition has been widely discussed on the Portuguese public health agenda, with Portugal taking important steps towards the implementation of a food and nutrition policy. These steps include a concerted set of actions aiming at (positively) influencing the nutritional status of a given population, carried out by representative institutions and by those who legitimately represent the communities for which the actions are intended [41, 42].

However, hard work needs to be done in order to support the development of preventive measures such as creating healthier food environments and empowering citizens towards healthy eating. If we consider that only 1.1% of the total healthcare budget in Portugal is spent on prevention, it is clearly understandable that there is a need for reinforcement of investment in this area [43]. A higher investment in prevention is essential to boost health gains, to increase the number of healthy years of life and to reduce health expenditure. A large part of this work can be initiated by the health sector, yet most determinants of healthy eating fall outside of the health sector and require intensive and intersectorial interventions.

### The challenge of creating a real intersectorial programme

The PNPAS, implemented in 2012, was developed under the scope of the Ministry of Health, Since the beginning of its implementation, the PNPAS has identified that several determinants of dietary intake were outside the control and influence of the health sector, requiring a more comprehensive and whole-of-government approach. Since the implementation of PNPAS in 2012, some of the initiatives were already based on an intersectorial collaboration. For example, PNPAS closely collaborated with the Ministry of Education and with the Ministry of Social Affairs in order to improve the nutritional quality of food supplies at schools and in food aid programmes for low-income groups. However, these ad hoc alliances, coordinated by a single ministry, did not hold accountable all of the other sectors. In addition, they did not allow the identification of common goals where the responsibility of all to achieve them was perceived. Indeed, besides PNPAS and the health sector, several other government institutions have important responsibilities regarding food. For example, food labelling in Portugal falls under the mandate of the Ministry of Agriculture, Forestry and Rural Development, and food safety policy falls under two different ministries, namely the Ministry of Agriculture, Forestry and Rural Development and the Ministry of Economy. This poses important challenges for interventions in this area, which we tried to address with the publication of EIPAS in December 2017. Afterwards, there was a commitment from seven different ministries to develop and implement different strategies and interventions for the promotion of healthy eating.

Traditionally, health issues are a much smaller concern for some government sectors. Therefore, it will be important to identify interventions that have the capability to combine the interest of several ministries and identify economic and social gains that can be obtained with investments on prevention and promotion of healthy eating.

For example, the promotion of the Mediterranean diet, which is widely represented along the 51 actions of EIPAS, is a transversal area of work that can aggregate the interest of almost all government sectors, namely agriculture, sea, education, economy, tourism, environment and culture, besides health.

Moreover, more collaborative work is needed with the agriculture, sea and environment sector in order to promote short food supply chains, nutrition-sensitive agriculture and fishing practices. For example, the promotion of the consumption of organic food through the National Strategy for Organic Production is one of the areas of intervention, in collaboration with the agriculture sector, that was approved in 2017 and which will be addressed within the EIPAS.

The ‘health in all policies’ approach is necessary, but it will bring new challenges and difficulties. The ‘whole-of-government’ approach should not be seen as a loss of power and responsibilities by the health sector, but as a way to empower and collaborate side by side with other stakeholders for the objectives of a healthy society. Further challenges of this approach will be the participation of health professionals in areas outside the health sector, namely in leadership, advocacy, legislation and policy, where there is no tradition or experience of intervention, with the need to create long-term consensus with stakeholders of the food system whilst simultaneously dealing with 4 year policy cycles.

### The challenge of obtaining quality information for decision-making

In order to develop the EIPAS proposal and to start the process, it was essential to have access to quality information to identify areas of greater risk, determinants of food consumption, and a history of initiatives and activities that promoted healthy eating. In particular, best practices as well as unsuccessful cases and their causes were reflected upon. Additionally, PNPAS released an annual report (data collected between 2012 and 2016) with the most recent data on food and nutrition in Portugal and a critical reflection about the work done by several stakeholders. PNPAS has also created the conditions for the development of the second national food intake survey, after a period of more than 30 years without institutional and comparable information regarding the food consumption of the Portuguese population. PNPAS integrates the WHO European Childhood Obesity Surveillance Initiative, being a team partner responsible for its development in Portugal [3]. Several other studies were carried out with the administrative, financial and scientific support of PNPAS. With support from WHO, one study evaluated the presence of trans fatty acids in foods sold in Portugal [44] and another evaluated the knowledge and capacity of the Portuguese population to understand and use nutritional labelling [36]. Moreover, during the economic crisis in Portugal, PNPAS implemented a system for monitoring and evaluating household food insecurity in the Portuguese population [45]. We believe that all the published data regarding the situation of food and nutrition in Portugal has greatly contributed to the policy decision-making and to placing nutrition in the public health and political agenda. In terms of surveillance systems on food and nutrition, these complex and expensive data collecting systems are being presented in an easily applicable format and more frequently developed to ensure the capacity to monitor food intake, and to evaluate trends in the nutritional composition of foods available in the market. Monitoring the nutritional profile of foods available in the market, and in particular those with the greatest market share, will allow us to understand how changes in food supply might be affecting the consumption and nutrient intake of individuals. Other countries, such as France, have already implemented monitoring systems of the nutritional profile of foods [46]. This will be particularly important to target the EIPAS ongoing strategy on food reformulation and on monitoring food industry self-regulation agreements. Under the scope of the new strategy, we are now designing a methodology for that purpose.

The existence of high-quality information on food and nutrition is an important key for the policy decision-making. Over the last decades, the lack of data in this area, in particular data on national food consumption over the last 30 years in Portugal, has been used as an argument for the lack of decision-making. However, to date, food sector companies are likely those with the best knowledge of consumer food behaviour. To our knowledge, one of the main challenges for EIPAS is to contribute to the building of conditions that will allow the regular collection of quality data for better informed decision-making (strengthening research on nutrition), as well as monitoring and evaluating the impact of the implemented actions. It will be crucial to make an investment on the evaluation of the impact of these different kinds of actions to better understand and identify the most successful and cost-effective interventions.

Indeed, there is limited scientific evidence available in terms of the effectiveness of the different policy approaches for nutrition interventions. Additionally, most of the cost-effectiveness studies have not shown even food environment-focused strategies to be effective on improving dietary habits and on obesity prevention [47, 48]. However, there is evidence that regulatory approaches are more effective [49–51] and, on the other hand, other studies have shown that this kind of interventions might have a positive impact in other indicators. For instance, strategies to improve nutrition labelling and food taxes can be an incentive to promote nutritional reformulation by the food industry [49, 52, 53].

### The challenge of changing food environments

The new Portuguese strategy on food and nutrition has been progressively using a more regulatory approach to promote the creation of healthier food environments. The more frequent regulatory approach in public health policies has raised discussions in public opinion, namely the implementation of those related to food availability in healthcare public institutions. Nevertheless, there is consistent evidence that suggests that regulatory strategies are efficient and cost-effective [54]. Public settings, like schools or health units, are places where healthy eating is being promoted, and therefore it is contradictory to offer food of poor nutritional quality there. Additionally, improving food availability in public facilities and other settings offers a great opportunity to improve the dietary habits of a population, in particular in those institutions that provide food for vulnerable groups such as schools, healthcare facilities and care institutions for the elderly. Therefore, ensuring availability of healthy food choices will lead to an increase in interest, particularly considering that, in recent years, unhealthy food has been widely available in these settings. In the future, interventions in this field should consider other target institutions such as the social institutions that support the elderly.

Changing and regulating food availability (namely through taxation) cannot be separated from food reformulation. In many cases, regulation encourages food product reformulation. From our experience gained with the sugar tax on sweetened beverages in Portugal, we can conclude that it was essential to encourage food reformulation of this category of food products. On the other hand, it increased interest in self-regulation agreements by the food industry and other food sectors. In the future, it will be important to study the impact of these approaches.

In addition to sugar, salt intake was also addressed in the Portuguese strategy on food reformulation. In Portugal, high salt intake is considered one of greatest public health issues. The Portuguese population consumes, on average, 10.7 g of salt per day [55], which is twice as much as the WHO recommendation. According to the Global Burden Disease study, a high intake of salt is the dietary risk factor that most contributes to the burden of disease in Portugal [1]. The estimated prevalence of hypertension in Portugal is 36% [6] and cardiovascular diseases are the leading cause of death [1]. Moreover, the investment that is been done with regards to salt reduction initiatives has the capacity to contribute to important health gains. WHO has considered that salt reduction initiatives are the ‘best buy’ interventions for the prevention of chronic diseases, suggesting that decreasing salt intake to less than 5 g per day might reduce the risk of stroke by 23% and the overall risk of cardiovascular diseases by 17% [56]. Nevertheless, the use of salt in food preparation and cooking is probably one of the highest contributors of salt intake in the Portuguese population. Data from the national survey on food and nutrition showed that soup is the dish that contributes the highest to salt intake. Considering this data, we developed a proposal for a voluntary agreement with the Portuguese Association of the Catering Sector, where maximum levels for salt in soup and main dishes were defined [57].

The creation of healthier food environments requires a set of interventions to change the food supply, which includes interventions to promote the reformulation of the nutritional composition of foods, as well as interventions to change food availability in several public settings. This is likely one of the most sensitive areas of intervention due to pressures of the economic sector when interventions to limit the food supply are implemented.

### The challenge of communicating with all and being effective in an era of social networks

In combination with these regulatory policies, it is important to implement communication campaigns to raise awareness and to empower citizens to make healthier food choices. Therefore, in 2018, we will also work in the development of mass media and social marketing campaigns. A voluntary agreement was established with the main Portuguese TV channels. Television will be used as a media tool for these healthy eating promotion campaigns in order to enable the exposure to messages to large populations. During the last years, PNPAS has begun playing a more active role in the communication and dissemination of accurate information on food and nutrition. Several educational materials have been published, both for the general population as well as for healthcare and other professions. Furthermore, the usage of new technologies and communication channels (digital/social media) are now widely used by PNPAS [13]. PNPAS is also trying to transform its communication networks into a reference for the general population to search for information on food and nutrition. In this area, we identified two main concerns, namely regarding how to reach large populations and the best strategies available. Moreover, it is important to consider the need to reach the most disadvantaged groups. PNPAS has been using innovative approaches for this purpose, for example, it developed and implemented a Massive Open Online Course on healthy eating, reaching more than 8000 participants throughout two editions. Additionally, PNPAS developed materials for specific vulnerable population groups (e.g. Mediterranean diet information in braille for blind persons).

One of the main challenges of EIPAS will be the creation of conditions to promote the dissemination of information on food and nutrition in several public institutions that is independent from the economic interests. The dissemination of this information in public institutions will allow us to reach large populations, namely the most vulnerable, with the same intensity of communication in social media.

### The challenge of acting locally

EIPAS recognised the importance of municipalities in the promotion of healthy eating. Therefore, local authorities will be the key actors in implementing part of EIPAS actions. A growing level of political decentralisation to local authorities has been observed in Portugal over the last years. Municipalities have the advantage of being closer to the population, enabling a better knowledge of local needs and intersectorial interventions. Thus, EIPAS will provide technical support for the implementation of its actions at the local level by the municipalities and primary healthcare centres.

One EIPAS challenge will be the protection of traditional foods and local traditions that add value to a healthy diet and, at the same time, promote local employment and biodiversity. However, due to old traditions of food preservation using salt and smoke, not all traditional products can be promoted, requiring discussion and compromise between all parties involved. Another important aspect is the need to promote seasonal and locally produced foods, which require less transportation and packaging, yet without creating barriers to free competition between European countries or other countries, which also deserves careful discussion in the implementation of these actions.

Certain measures can be implemented to address this issue, for example, we have already initiated a mapping of all the municipality initiatives on food and nutrition and the National Programme for the Promotion of Healthy Eating is now implementing partnerships with several Portuguese municipalities, supporting them in the implementation of a local strategy based on the EIPAS principles.

### The challenge of formation

There is a need for investment in professional human resources regarding nutrition, not only for health professionals but also for other professionals that might influence food consumption, namely, teachers, social care professionals and food service personnel (canteen employees).

The growing interest in healthy eating has led to a problem of misinformation on nutrition issues. EIPAS should promote and encourage the dissemination of evidence-based nutrition information for health and other professionals. Moreover, due to an increasing interest and participation of different professionals in public health nutrition issues, leadership skills should be considered as a key factor in supporting actions to promote healthy eating. These skills will be decisive for the implementation of the EIPAS strategy in different settings such as local authorities and primary healthcare centres. Leadership development will be an important demand for the future intersectorial actions on nutrition in order to change food environments. Further, these skills should also be ensured in the curricula of health professionals.

### The challenge of the future – integrating environmental sustainability and culture

Despite the interministerial approach of EIPAS, we believe that other sectors and stakeholders should also be involved in this strategy. Additionally, environmental sustainability should also be progressively integrated with food and nutrition policies. The need to obtain more sustainable food systems is a current challenge that should also be addressed through an integrated strategy on food and nutrition. Concerns about climate change and environmental sustainability are present in the international political agenda and should be considered the two biggest challenges facing society today. Food choices have a large impact on the environment and climate change, yet it is known that recommendations for a healthy diet can be combined with those for sustainable diets [58]. As an example to address this issue, EIPAS is working with the Ministry of Agriculture and the Portuguese strategy for the promotion of organic foods in order to develop guidance to increase the utilisation of these foods in public canteens; this issue could be addressed in public food procurements.

Another important debate involving food policies and cultural policies needs to be encouraged. Since the Mediterranean diet is an eminent cultural heritage and framed in a certain way of life, it is necessary to increase the involvement of cultural actors in their anthropological and cultural cataloguing, as well as in their cultural production at various levels and dimensions.

## Conclusions

For the first time, Portugal has a nutrition policy based on the WHO concept of ‘health in all policies’ and on national data regarding food and nutrient intake. For the following years, an interministerial working group will promote the collaboration between the different ministries and will follow-up EIPAS operationalisation. The capacity to implement all of the 51 actions of EIPAS and the inherent difficulties that will arise will make this process an authentic political and social laboratory that deserves to be carefully monitored.

## Abbreviations

EIPAS: Integrated Strategy for the Promotion of Healthy Eating
PNPAS: National Programme for the Promotion of Healthy Eating
WHO: World Health Organization

## References

[1] Direção-Geral da Saúde, Direção de Serviços de Informação e Análise (2017). A Saúde dos Portugueses 2016.

[2] Lopes C, Torres D, Oliveira A, Severo M, Alarcão V, Guiomar S, Mota J, Teixeira P, Rodrigues S, Lobato L (2017). Inquérito Alimentar Nacional e de Atividade Física IAN-AF 2015–2016. Relatório de resultados.

[3] Rito A, RCd S, Mendes S, Graça P (2017). Childhood Obesity Surveillance Initiative. COSI Portugal 2016.

[4] Redon J, Olsen MH, Cooper RS, Zurriaga O, Martinez-Beneito MA, Laurent S, Cifkova R, Coca A, Mancia G (2011). Stroke mortality and trends from 1990 to 2006 in 39 countries from Europe and Central Asia: implications for control of high blood pressure. European Heart Jounal.

[5] Instituto Nacional de Estatística. Rendimento e Condições de Vida 2017 (Dados provisórios). 2017. Available: https://www.ine.pt/xportal/xmain?xpid=INE&xpgid=ine_destaques&DESTAQUESdest_boui=281441156&DESTAQUESmodo=2. Accessed 29 May 2018.

[6] Barreto M, Gaio V, Kislaya I, Antunes L, Rodrigues AP, Silva AC, Vargas P, Prokopenko T, Santos AJ, Namorado S (2016). 1° Inquérito Nacional de Saúde com Exame Físico (INSEF 2015): Estado de Saúde.

[7] Lopes C, Torres D, Oliveira A, Severo M, Alarcão V, Guiomar S (2017). Anexo 3 - Contributos dos alimentos para a ingestão nutricional.

[8] do Carmo I, Dos Santos O, Camolas J, Vieira J, Carreira M, Medina L, Reis L, Myatt J, Galvao-Teles A (2008). Overweight and obesity in Portugal: national prevalence in 2003-2005. Obesity Rev.

[9] Diário da República 2.ª série – N.° 10 – 13 de janeiro de 2012: Despacho n.° 404/2012. 2012. Available: https://dre.pt/web/guest/pesquisa/-/search/2150516/details/normal?q=Despacho+n.°%20404%2F2012+13+de+janeiro. Accessed 19 May 2018.

[10] Programa Nacional para a Promoção da Alimentação Saudável, Direção-Geral da Saúde (2012). Programa Nacional para a Promoção da Alimentação Saudável - Orientações Programáticas.

[11] Graça P, Gregório MJ (2013). The Construction of the National Program for the Promotion of Healthy Eating - Conceptual Aspects, Strategic Guidelines and Initial Challenges. Revista Nutrícias.

[12] Diário da República. 2.ª série – N.° 249 – 29 de dezembro de 2017: Despacho n.° 11418/2017. Estratégia Integrada para a Promoção da Alimentação Saudável. 2017. Available: https://dre.pt/pesquisa/-/search/114424591/details/normal?l=1. Accessed 19 May 2018.

[13] Graça P, Gregório MJ, SMd S, Carriço J, Correia A, Salvador C (2016). The Portuguese National Programme for the Promotion of Healthy Eating: 2012-2015. Public Health Panorama.

[14] Presidência do Conselho de Ministros: DB 334/2016. Deliberação do Conselho de Ministros, que cria um Grupo de Trabalho para a elaboração de uma estratégia para a promoção da alimentação saudável. Lisbon: Presidência do Conselho de Ministros; 2016.

[15] Santos JL, Id C, Graça P, Ribeiro I (2013). The Future of Food: Environment, Health and Economy.

[16] Raine KD, Atkey K, Olstad DL, Ferdinands AR, Beaulieu D, Buhler S, Campbell N, Cook B, L’Abbé M, Lederer A (2018). Healthy food procurement and nutrition standards in public facilities: evidence synthesis and consensus policy recommendations. Health Promotion and Chronic Disease Prevention in Canada - Research, Policy and Practice.

[17] Lima RM. Orientações sobre ementas e refeitórios escolares. Direção-Geral de Educação. Lisboa, Portugal. 2018. Available: http://www.dge.mec.pt/sites/default/files/Esaude/oere.pdf. Accessed 21 Oct 2018.

[18] Ladeiras L, Lima RM, Lopes A (2012). Bufetes escolares – orientações.

[19] Diário da República 2.a série – N.o 248 – 28 de dezembro de 2017: Despacho n.° 11391/2017. 2017. Available: https://dre.pt/home/-/dre/114412574/details/2/maximized. Accessed 4 May 2018.

[20] Diário da República 2a série no 108 de 6 de junho de 2016: Despacho n.° 7516-A/2016. 2016. Available: https://dre.pt/pesquisa/-/search/74604818/details/normal?l=1. Accessed 4 May 2018.

[21] Graça P, Gregório MJ, Santos A, SMd S (2016). Redução do Consumo de Açúcar em Portugal: Evidência que Justifica Ação.

[22] Diário da República n° 248/2016 Série I de 2016-12-28: Lei 42/2016 - Orçamento do Estado para 2017. Secção III. Impostos especiais de consumo. Artigo 211.° Imposto sobre o álcool, as bebidas alcoólicas e as bebidas adicionadas de açúcar ou outros edulcorantes (IABA). 2016. Available: https://dre.pt/home/-/dre/105637672/details/maximized?serie=I&dreId=105637665. Accessed 4 May 2018.

[23] Goiana-da-Silva Francisco, Nunes Alexandre Morais, Miraldo Marisa, Bento Alexandra, Breda João, Araújo Fernando Ferreira (2018). Fiscalidade ao Serviço da Saúde Pública: A Experiência na Tributação das Bebidas Açucaradas em Portugal. Acta Médica Portuguesa.

[24] Gregório MJ, Rodrigues AM, Graça P, RDd S, Dias SS, Branco JC, Canhão H (2018). Food insecurity is associated with low adherence to the Mediterranean Diet and adverse health conditions in Portuguese adults. Front Public Health.

[25] Neter JE, Dijkstra SC, Dekkers ALM, Ocke MC, Visser M, Brouwer IA. Dutch food bank recipients have poorer dietary intakes than the general and low-socioeconomic status Dutch adult population. Eur J Nutr. 2017. 10.1007/s00394-017-1540-x. 10.1007/s00394-017-1540-x PMC6267415 28975454

[26] Gregório MJ, Tavares C, Cruz D, Graça P (2017). Programa de distribuição de alimentos: considerações para a adequação nutricional da oferta alimentar.

[27] Gregório MJ, Graça P (2017). Manual de orientação para a utilização adequada do cabaz de alimentos do Programa Operacional de Apoio às Pessoas mais Carenciadas (PO APMC) 2014–2020.

[28] Bernardino F, SMd S, Fernandes I, Gregório MJ, Graça P (2018). Alimentos Fornecedores de Proteínas no Cabaz de Alimentos do POAPMC.

[29] High Level Group on Nutrition and Physical Activity, European Commission: EU Framework for National Salt Initiatives. Available: http://ec.europa.eu/health/ph_determinants/life_style/nutrition/documents/salt_initiative.pdf. Accessed 5 May 2018.

[30] High Level Group on Nutrition and Physical Activity, European Commission: Annex II: Added Sugars - EU Framework for National Initiatives on Selected Nutrients. Available: https://ec.europa.eu/health//sites/health/files/nutrition_physical_activity/docs/added_sugars_en.pdf. Accessed 5 May 2018.

[31] High Level Group on Nutrition and Physical Activity, European Commission: EU Framework for National Initiatives on Selected Nutrients. Available: https://ec.europa.eu/health//sites/health/files/nutrition_physical_activity/docs/euframework_national_nutrients_en.pdf. Accessed 5 May 2018.

[32] Public Health England (2017). Salt Reduction Targets for 2017.

[33] Public Health England (2017). Sugar Reduction: Achieving the 20%. A Technical Report Outlining Progress to Date, Guidelines for Industry, 2015 Baseline Levels in Key Foods and Next Steps.

[34] World Health Organization (2015). Eliminating Trans Fats in Europe. A Policy Brief.

[35] Diário da República n° 155/2009 1ª Série de 12 de agosto de 2009: Lei n.° 75/09, Estabelece normas com vista à redução do teor de sal no pão bem como informação na rotulagem de alimentos embalados destinados ao consumo humano. 2009. Available: https://dre.pt/pesquisa/-/search/493513/details/maximized. Accessed 29 May 2018.

[36] Gomes S, Nogueira M, Ferreira M, Gregório MJ (2017). Portuguese Consumers’ Attitudes Towards Food Labelling.

[37] Grupo Parlamentar Bloco de Esquerda (2017). Projeto de Resolução N.° .../XIII/3.ª - Recomenda ao Governo a inclusão do semáforo nutricional nos alimentos embalados.

[38] Representação Parlamentar PAN (2018). Projecto de Resolução n.o 1297/XIII/3a - Recomenda ao Governo que inclua o sistema de Semáforo Nutricional e do Semáforo Carcinogénico na declaração nutricional obrigatória constante nos alimentos embalados.

[39] Diário da República n° 65/2018 Série I de 2018-04-03: Resolução da Assembleia da República n.° 83/2018 - Recomenda ao Governo que avalie, defina e implemente formas complementares de informação sobre o teor nutritional dos alimentos. 2018. Available: https://dre.pt/home/-/dre/114949211/details/maximized. Accessed 7 May 2018.

[40] WHO European Action Network on Reducing Marketing Pressure on Children (2016). Report of 11th meeting in Lisbon, Portugal, 21–22 April 2016.

[41] Helsing E (1997). The history of nutrition policy. Nutr Rev.

[42] Graça P., Gregório M.J., Mendes de Sousa S., Camolas J. (2016). Food Policy in Portugal—Historical Context, Opportunities, and Threats. Reference Module in Food Science.

[43] CNS - Conselho Nacional de Saúde: Fluxos financeiros do SNS. 2017. http://www.cns.min-saude.pt/wp-content/uploads/2017/09/Fluxos_Financeiros_SNS_3.11.2017_final.pdf. Accessed 3 May 2018.

[44] Casal S, Cruz R, Costa N, Graça P, Breda J (2016). Trans-Fatty Acids in Portuguese Food Products.

[45] Gregório MJ, Graça P, Santos AC, Gomes S, Portugal AC, Nogueira PJ: Relatório INFOFAMÍLIA 2011–2014–Quatro anos de monitorização da Segurança Alimentar e outras questões de saúde relacionadas com condições socioeconómicas, em agregados familiares portugueses utentes dos cuidados de saúde primários do Serviço Nacional de Saúde 2011–2014*.* Lisboa: Direção-Geral da Saúde; 2017.

[46] Dunford Elizabeth, Webster Jacqui, Metzler Adriana Blanco, Czernichow Sebastien, Mhurchu Cliona Ni, Wolmarans Petro, Snowdon Wendy, L’Abbe Mary, Li Nicole, Maulik Pallab K, Barquera Simon, Schoj Verónica, Allemandi Lorena, Samman Norma, de Menezes Elizabete Wenzel, Hassell Trevor, Ortiz Johana, de Ariza Julieta Salazar, Rahman A Rashid, de Núñez Leticia, Garcia Maria Reyes, van Rossum Caroline, Westenbrink Susanne, Thiam Lim Meng, MacGregor Graham, Neal Bruce (2011). International collaborative project to compare and monitor the nutritional composition of processed foods. European Journal of Preventive Cardiology.

[47] Storcksdieck Genannt Bonsmann S, Wills JM (2012). Nutrition labeling to prevent obesity: reviewing the evidence from Europe. Current Obesity Reports.

[48] Falbe J, Thompson HR, Becker CM, Rojas N, McCulloch CE, Madsen KA (2016). Impact of the Berkeley excise tax on sugar-sweetened beverage consumption. Am J Public Health.

[49] Mozaffarian D, Angell SY, Lang T, Rivera JA (2018). Role of government policy in nutrition-barriers to and opportunities for healthier eating. BMJ.

[50] Afshin A, Penalvo J, Del Gobbo L, Kashaf M, Micha R, Morrish K, Pearson-Stuttard J, Rehm C, Shangguan S, Smith JD (2015). CVD Prevention Through Policy: a Review of Mass Media, Food/Menu Labeling, Taxation/Subsidies, Built Environment, School Procurement, Worksite Wellness, and Marketing Standards to Improve Diet. Current cardiology reports.

[51] Mozaffarian D, Afshin A, Benowitz NL, Bittner V, Daniels SR, Franch HA, Jacobs DR, Kraus WE, Kris-Etherton PM, Krummel DA (2012). Population approaches to improve diet, physical activity, and smoking habits: a scientific statement from the American Heart Association. Circulation.

[52] Young I, Waddell L, Harding S, Greig J, Mascarenhas M, Sivaramalingam B, Pham MT, Papadopoulos A (2015). A systematic review and meta-analysis of the effectiveness of food safety education interventions for consumers in developed countries. BMC Public Health.

[53] Briggs ADM, Mytton OT, Kehlbacher A, Tiffin R, Elhussein A, Rayner M, Jebb SA, Blakely T, Scarborough P (2017). Health impact assessment of the UK soft drinks industry levy: a comparative risk assessment modelling study. Lancet Public Health.

[54] Shill J, Mavoa H, Allender S, Lawrence M, Sacks G, Peeters A, Crammond B, Swinburn B (2011). Government regulation to promote healthy foodenvironments – a view from inside state governments. Obes Rev.

[55] Polonia Jorge, Martins Luis, Pinto Fernando, Nazare Jose (2014). Prevalence, awareness, treatment and control of hypertension and salt intake in Portugal. Journal of Hypertension.

[56] World Health Organization Regional Office for Europe (2016). Action Plan for the Prevention and Control of Noncommunicable Diseases in the WHO European Region.

[57] Proposta de Estratégia para a redução do consumo de sal na população portuguesa através da modificação da disponibilidade da oferta. Grupo de trabalho criado pelo Despacho n.° 8272/2015, de 29 de julho, e constituído pelas seguintes Entidades: Direção-Geral da Saúde (coordenador), Associação da Hotelaria, Restauração e Similares de Portugal (AHRESP), Associação Portuguesa de Empresas de Distribuição (APED), Autoridade da Segurança Alimentar e Económica (ASAE), Confederação de Comércio e Serviços de Portugal (CCP), Associação Portuguesa para a Defesa do Consumidor (DECO), Direção-Geral das Atividades Económicas (DGAE), Direção-Geral de Alimentação e Veterinária (DGAV), Direção-Geral do Consumidor (DGC), Federação das Indústrias Portuguesas Agro-Alimentares (FIPA) e Portugal Foods. Lisbon: Direção-Geral da Saúde; 2015.

[58] Macdiarmid JI (2013). Conference on ‘Future food and health’ Symposium I: Sustainability and food security - Is a healthy diet an environmentally sustainable diet?. Proc Nutr Soc.

[59] Regulamento (CE) n.o 1924/2006 do Parlamento Europeu e do Conselho, de 20 de dezembro de 2006, relativo às alegações nutricionais e de saúde sobre os alimentos. Available: https://eur-lex.europa.eu/legal-content/PT/TXT/PDF/?uri=CELEX:32006R1924&from=PT. Accessed 2 May 2018.

[60] World Health Organization (2015). Fact Sheet N.o 394: Healthy Diet.

[61] World Health Organization (2015). Guideline: Sugars Intake for Adults and Children.

[62] World Health Organization. European Food and Nutrition Action Plan 2015–2020. Copenhagen: WHO Regional Office for Europe; 2015.

[63] High Level Group on Nutrition and Physical Activity, European Commission (2014). EU Action Plan on Childhood Obesity 2014–2020.

